# Language of Hope in Conversations between Pediatric Oncologists, Children with Advanced Cancer, and their Families

**DOI:** 10.21203/rs.3.rs-10617761/v1

**Published:** 2026-09-30

**Authors:** Nidhi Vij Mali, Carolyn Humphrey, Amy Porter, Erica Carmen Kaye, Alexandra Kathleen Superdock

**Affiliations:** St. Jude Children’s Research Hospital; University of Mississippi; Massachusetts General Hospital; St. Jude Children’s Research Hospital; St. Jude Children’s Research Hospital

**Keywords:** Communication, Hope, Pediatric Cancer, Parents, Breaking Bad News, Goals of Care

## Abstract

**Purpose:**

Hope is a key driver of coping and decision-making for children with cancer and their families. Prior studies have shown that hope is bolstered, not fractured, by high-quality prognostic communication. However, clinicians describe a tension between conveying hope and honestly discussing poor prognosis. This prospective longitudinal observational study aimed to identify and characterize communication approaches used by oncologists and parents to navigate hope in the context of poor prognosis.

**Methods:**

Serial conversations between pediatric patients with poor-prognosis brain tumors, their families, and their medical teams were audio-recorded at disease reevaluation timepoints for up to 24 months or until death. Rapid qualitative analysis was used to characterize the frequency and content of hope-related dialogue.

**Results:**

132 conversations were recorded for 30 brain tumor patient-parent-oncologist triads.

Hope-related dialogue was identified in 73 (55%) of encounters. Oncologists introduced hope language in about two-thirds of these conversations. Hope dialogue could be described along two dimensions: temporal orientation and desired outcomes. Temporally, hope appeared in conversations as prospective, retrospective, or negated. Desired outcomes included hope for cure, hope for remission, hope for life prolongation and meaningful experiences, and hope for symptom management and comfort at the end of life.

**Conclusion:**

Hope dialogue may be an entry point for aligning with families, exploring prognostic understanding, and opening the door to deeper conversations about reframing hopes and goals of care. Future work should explore development of strategies to elicit and respond to patient and family hopes across the cancer trajectory.

## Introduction

Prognostic communication is an essential component of comprehensive care for children with cancer [1]. Provision of information about treatment progress, chance of cure, and long-term impact helps patients and families make decisions and evolve expectations for the future [2]. Patients and parents value honest communication that is both realistic and hopeful [3–5], yet many cancer care professionals say it is challenging to support hope when prognosis is poor [3, 6, 7]. Some clinicians fear that direct discussion of poor prognosis will shatter hope or that affirmation of hope will lead to unrealistic expectations [8, 9]. However, research shows that parents can hold accurate prognostic awareness while still maintaining hope and that parents who heard clear, detailed prognostic disclosure actually reported higher levels of hope, even if survival was unlikely [8, 10].

‘Hope’ is a complex multidimensional construct influenced by culture, spirituality, psychology, and social context [11]. In the setting of pediatric cancer, hope can support adaptive coping [12–14], enhance quality of life [15], and protect against adverse psychosocial outcomes for patients and families alike [16, 17]. Families often cite hope as a factor that drives decisions about treatment and goals of care [13]. When prognosis is poor, families may hold a multitude of hopes, encompassing hopes for cure as well as hopes related to life prolongation, comfort, meaningful experiences, and legacy [18–22]. Although the tenor and target of hope evolve throughout the cancer trajectory, hope endures as a meaningful part of the patient-family experience, even at the end of life and beyond [8, 23, 24]. The National Cancer Institute advises clinicians to discuss and support hope throughout the cancer trajectory, not only to ensure compassionate care, but also to improve health outcomes, optimize goal-concordant care, and relieve suffering [25].

Despite growing literature describing the influence of hope on cancer care and communication, little is known about how children, parents, and oncologists articulate hope during prognostic discussions. Existing research on the role of hope in clinical communication largely relies on cross-sectional, retrospective accounts from caregivers and clinicians, raising concerns about recall and social desirability biases [26]. To address this gap, this longitudinal observational study aimed to characterize the frequency and content of hope-related dialogue during disease reevaluation discussions among a sample of children with poor-prognosis brain tumors. By learning how oncologists, patients, and families communicate about hope in real-time, we can develop approaches to support clinicians in assessing and responding to patient and family hope as cancer advances.

## Methods

Understanding Communication in Healthcare to Achieve Trust (UCHAT) is an IRB-approved, prospective, longitudinal study of the evolution of communication in advancing childhood cancer (NCT02846038, St Jude IRB- Pro00006473). This protocol was co-designed in partnership with patients, caregivers, and multidisciplinary clinicians [27,28]. Study conduct and presentation of findings were informed by the five core principles of OnQual (engagement, flexibility, transparency, transferability, impact), comprising expert recommendations for rigor in the conduct of qualitative science in oncology [29]. OnQual and COREQ reports are provided in Supplements 1–2.

Details regarding study design co-creation with patients and families, eligibility criteria, recruitment, enrollment, informed consent, and data collection processes have been previously described [26,27]. This sub-analysis focused on the subset of patients with poor-prognosis central nervous system (CNS) tumors, as this population included patients with incurable disease at diagnosis who often experienced clear disease progression and clinical decline, generating fertile ground for exploration of hope language in the setting of poor-prognosis cancer.

Patients were eligible if they were £ 30-years-old and diagnosed with a poor-prognosis brain tumor, defined as survival odds £ 50% as estimated by their primary oncologist. For each participating patient, one caregiver was enrolled. Caregivers were eligible if they were ^3^ 18-years-old, had English proficiency, and planned to accompany the patient to medical visits. For each participating patient, the oncologists who provided primary, longitudinal cancer care to the patient at the study institution were eligible. All participants provided consent for the clinical encounters to be audio recorded. Other individuals who were present during recorded encounters (e.g. other clinicians or family members) also consented to be recorded but were not included in the study population.

Data collection consisted of audio-recording medical encounters between pediatric patients with poor-prognosis cancer, families, and clinicians at serial time points of disease reevaluation. Patients were followed until the child’s death or 24 months following relapse, whichever occurred first. Disease reevaluation visits were defined as any clinic or inpatient discussion including disclosure of results from a diagnostic test (e.g. biopsy, imaging, tumor biomarkers, etc.) to assess disease status. For each encounter during the study period, the primary oncologist recorded the conversation using a portable audio-recording device and categorized the disease status as 1) progression, 2) regression or stable disease, or 3) equivocal (i.e. progression and regression were both identified, usually for multifocal disease). For this analysis, we used disease status categorization to assess the context of identified hope dialogue.

Our research team used Rapid Qualitative Analysis (RQA) [30] to generate structured summaries of hope-related language within and across disease reevaluation encounters, recognizing RQA is a rigorous methodology for synthesizing large-volume data with the goal of efficient application of findings to inform educational and clinical advancements [31,32]. RQA procedures for other analyses from this study have been previously described [33,34]. Briefly, two members of the research team (EK, AP) drafted a template for longitudinal episode summaries which included a dedicated section for any language related to hope that occurred during a recorded encounter (Supplement 3). The research team reviewed and revised this template to define communication about hope as any statements made by patients, parents, or clinicians that explicitly referenced “hope” or implicitly referenced desired possibilities and expectations for the future.

Using this template, analysts (AP, EC, JN) reviewed longitudinal sets of recorded encounters and completed episode summaries, documenting whether hope language occurred, what was articulated, who initiated it, and how it evolved over time (if at all). Half of the recordings were reviewed and summarized by two independent analysts and then reviewed by a third party (EK) to ensure consistency; once analysts demonstrated consistency in summary content, individual analysts summarized the remainder of the recordings. Two members of the team (NVM, CH) then reviewed the episode summaries and compiled quotes and descriptions from episode summaries into a matrix, where rows represented patients and columns represented sequential recorded encounters. The analysis team (NVM, CH, EKC, AKS) conducted multiple rounds of memo-writing, identified key concepts, and synthesized concepts to create a multidimensional descriptive framework for hope-related dialogue identified during recorded encounters.

## Results

We analyzed 132 disease reevaluation conversations, totaling more than 2,400 minutes of recorded dialogue between 30 distinct pediatric patient-parent-oncologist triads. Table 1 presents demographic data for participants. Hope-related language appeared in 73 of the 132 conversations (55%). Oncologists initiated or introduced hope-related language in 50 conversations (38% of all conversations, 68% of conversations with hope language). Patients or parents initiated hope language in 10 conversations (7.6% of all conversations, 14% of conversations with hope language). In the remaining conversations where hope was discussed, the concept of hope emerged through dialogue exchange between different participants. About half (34, 47%) of discussions with hope dialogue occurred in the setting of disease progression, though disease progression conversations only represented one-third (43, 33%) of all recorded encounters.

RQA of the 73 conversations with hope-related dialogue generated an inductively derived framework comprised of themes organized within two dimensions: 1) temporal orientation and 2) desired outcomes (Table 2). ‘Temporal orientation’ described *when* hope occurred relative to the present discussion. ‘Desired outcomes’ described *what* the speaker hoped for. Exemplary quotes for both dimensions are presented in Table 3 and Table 4. Additionally, longitudinal data collection enabled observation of how hope language evolved over time within triads. Due to heterogeneity of the longitudinal data, we did not identify discrete patterns in how hope dialogue changed over time, with regard to content, context, or frequency. Dialogue about hope was usually self-contained within the encounter and not specifically referenced or revisited in subsequent encounters.

### First dimension: Temporal Orientation

Three temporal orientations of hope dialogue were identified: prospective (hope for the future), retrospective (reflections on past hopes), and negated (hopes relinquished in response to new information). Prospective hopes were expressed by parents, patients, and oncologists across a range of future-oriented goals that varied over time and diagnoses. Often, prospective hopes were centered on desired clinical outcomes and aspects of quality of life. For example, one oncologist expressed hope that the patient’s treatment would remain effective, stating, “You still got tumor there, so there’s a reason why you’re hurting. I think it’s going in the right direction. We hope that the chemotherapy continues to work on it.” Similarly, a parent expressed simultaneous future hopes for life prolongation and treatment efficacy, saying, “We’ve had this conversation before, that I’m not going to stop fighting for her. That’s my hope: Time. Cure.” When discussing hopes for the future, clinicians often incorporated cautionary or qualifying statements, like “I do not want to give you any false promises” or “I’m not sure.” In contrast, parents were more likely to express hopes in emotional or spiritual ways, like drawing on faith, personal values, or the belief in miracles.

Retrospective hopes were expressed by both oncologists and parents, typically related to whether earlier or different decisions might have improved outcomes. These reflections often revealed unspoken or unfulfilled hopes or missed opportunities, particularly during periods of disease progression. One oncologist acknowledged the concept of unfulfilled hope, saying:

“I’m really, really sorry…You know, we kind of have this at the back of the mind that this disease is bad, but every time we do scans, we go in with the hope that this child is going to be different. And I was really hoping she was going to be different…. given how well she was looking.”

When oncologists expressed retrospective hopes, parents often inquired about alternative options, sometimes with initial disappointment, but then with acceptance of the situation, shifting the discussion toward planning quality time and next steps.

Hope dialogue was categorized as ‘negated’ when the oncologist was delivering information that rendered some hopes unrealistic or emphasized the limitations of what could be hoped for. Negated hope dialogue typically occurred during conversations in which oncologists communicated limited treatment efficacy or a poor prognosis, often in the context of upfront fatal diagnoses or diseases with limited treatment options. In some cases, oncologists directly ruled out a hope for cure or remission or labeled treatment as ineffective. One oncologist negated hope for cure by articulating the certainty of future progression,

“Unfortunately, these tumors will recur, will come back after a while, unfortunately that’s their nature. I don’t know how soon or how late… it is almost certain that it’s going to come back. The whole idea of doing this medicine, this clinical trial, is to see if we can delay that from happening.”

In these conversations, oncologists aimed to balance hope with honesty, encouraging families to consider hopes beyond cure:

“I’m not saying that we should give up, but I also want to make sure the expectations are realistic, and I do not want to sugarcoat…I just do not want to give you any false promises….I would say at the most, it might buy us some time, but I cannot promise you that. But at best that’s what I’m hoping for.”

Often, these types of statements created space for some parents to verbalize their understanding of prognosis and appreciation for the oncologist’s honesty. Some parents responded with statements about their enduring hope for a different outcome and their intent to maintain a curative goal of care.

### Second dimension: Desired Outcomes

Parents, patients, and oncologists discussed interrelated categories of desired outcomes, including cure, remission, life prolongation, meaningful experiences, symptom management, and comfort at the end of life. Hope for cure was often framed as idealized, sometimes reflecting hope against all odds that the patient would be cured and, at other times, looking forward to the discovery of a cure for future children. Some oncologists articulated specific hopes about pharmacological breakthroughs that might lead to curative therapies. Families likewise expressed hope that clinical trial therapy would lead to a cure for their child. One parent remarked,

“I think we’d all feel a little bit better doing something than not doing something. That we want to try to keep things status quo for as long as possible in hope that [my child’s] cure is out there… [She’s] making bracelets to raise money for [the hospital].”

Oncologists and parents also expressed hope for remission or disease stability while waiting for a medical breakthrough. As one oncologist remarked:

“The best we are hoping for, is to delay that tumor from coming back. If it delays to a point where new treatments become available at that time, then she could be eligible for those trials. And that is the whole idea of doing this medicine.”

Discussion about hope for disease stability and life prolongation was especially prominent when the patient’s diagnosis was considered terminal, such as diffuse pontine intrinsic glioma (DIPG). Often, these expressions of hope opened the door for further discussion about treatment goals and next steps.

Hope for life prolongation was also discussed as part of hope for the child to have meaningful experiences, such as enjoying a special occasion or experience. One parent expressed, “I think honestly our main concern is getting through the holidays.” This statement led to a discussion about options for the family to be home for Thanksgiving and Christmas. In another case, an adolescent patient expressed hope to attend his school homecoming as an important milestone, enabling collaborative decision-making to honor this goal. When hopes were explicitly verbalized, clinicians expressed a willingness to honor these hopes however possible.

In the context of disease progression when no further cancer-directed treatments were available, statements of hope shifted toward symptom management and comfort. Oncologists sometimes incorporated hope for comfort into their treatment recommendations: “Hopefully steroids [will]…be enough to take care of symptoms and calm this down and let us go on ahead.” While discussion around specific hopes for end of life were minimal, one parent expressed hope for a swift death, saying, “I don’t know if he’ll just turn really quick. That’s what I’m hoping for.”

Importantly, expressions of hope coexisted with prognostic disclosure. In conversations around terminal illness, some oncologists reframed hope to focus on non-curative goals, following prognostic disclosure with a wish for disease stability, symptom management, or meaningful time. For example, one oncologist stated:

“It’s extremely rare to have beyond 1 year or 18 months, less than 10% chance. He could be in that 10% who are into their second year, it’s possible… Some extra radiation could buy us some more time.”

This type of framing acknowledged a grim prognosis while still creating space for possible hopes to grow.

## Discussion

In this analysis of serial disease reevaluation discussions with pediatric patients with poor-prognosis CNS tumors, families, and oncologists, hope dialogue appeared in approximately half of recorded encounters. Parents and oncologists frequently aligned in their hopes for medical breakthroughs, which allowed clinicians to clarify expectations of experimental therapy without diminishing hope. When patients and parents verbalized specific hopes about life prolongation, meaningful experiences, and symptom management, oncologists asked additional questions, adjusted treatment plans, or shifted to discussing next steps. Our findings show how hope dialogue may serve as a valuable entry point for aligning with families, exploring prognostic understanding, and opening the door to deeper conversations about reframing hopes and goals of care.

Although our study demonstrates that oncologists are sometimes incorporating hope-related language into clinical conversations, this remains an inconsistent practice. The predominance of oncologist-initiated references to hope in this study sample likely reflects the nature of disease reevaluation discussions as oncologist-led, rather than oncologists’ inherent comfort discussing hope. Oncologists often approached hope cautiously, including caveats or qualifying statements (e.g., “I’m not sure” or “I don’t want to give you any false promises”), prior to discussing the possibility of future clinical trials. Historically, oncologists have faced challenges balancing prognostic disclosure with maintaining hope: on one hand, afraid that being direct will destroy hope; while on the other hand, afraid that using hope language will enable denial or ‘false’ hope [8]. Yet, we found hope language more prevalent among discussions about disease progression (versus stability or regression), underscoring the opportunity to better equip oncologists to thoughtfully use and respond to hope language across the disease trajectory.

A synergy between prognostic communication and hope language relies on clear provision of information, intentional word choice, and attention to nonverbal expression. Prior research suggests that pediatric oncologists sometimes avoid or delay direct communication about poor prognosis, emphasizing statements that ‘space-hold hope’ for cure without disclosure of key medical information [37, 35, 36]. Novel communication approaches have been proposed to equip oncologists for conveying hope alongside honest prognostic communication, including ‘seed-planting’ language, in which clinicians share worries about future disease progression during timepoints of relative stability, while also validating hopes and celebrating day-by-day wins with patients and families [33, 37]. Short phrases that can be easily incorporated into clinical communication to acknowledge hope across circumstances include “I share your hope. I also worry that…,” “Let’s hope for the best while preparing for the worst,” and “Whatever happens, we’ll have a plan.” [38–41] Patients and parents have also shared how the oncologist’s tone, facial expressions, and body language can impart reassurance and convey hope [40].

Within these data, diverse hopes were articulated that shifted across speakers, time, and clinical circumstances, affirming existing literature that characterizes hope in pediatric cancer as fluid, evolving, and ever-present [10, 24]. Given the dynamic nature of hope, it is imperative for clinicians to iteratively assess what patients and families are hoping for throughout the cancer trajectory. However, in this study, we identified no instances in which oncologists explicitly asked patients or parents about their hopes. Even when families spontaneously expressed their hopes and expectations, oncologists missed opportunities to deepen understanding through follow-up questions, like “What else are you hoping for?” or “What would that look like for you?” [42]. Such simple, open-ended prompts may elicit broader understanding of families’ hopes, goals, and priorities. Synder’s Hope Theory conceptualizes hope as a cognitive process involving goal-directed thinking, supported by agency (motivation to meet goals) and pathways (strategies to achieve goals) [43]. Consistent with Snyder’s theory, creating space for families to identify a range of valued goals may not only inform clinical decisions, but also cultivate empowerment and hopeful patterns of thinking [42, 43].

Adolescents and young adult patients used hope language infrequently during these discussions, likely for multifactorial reasons beyond the scope of this analysis; yet when they spoke, they more often expressed hope in relation to personal or experiential goals rather than in terms of clinical outcomes. These variations in frequency and manner of hope expression underscore the importance of empowering patients to share their hopes and wishes to ensure that all voices are heard and supported in care planning. Notably, prior research suggests that, among children and adolescents with cancer, hope is positively associated with health-related quality of life, in part through anxiety reduction [21]. In addition, narrative methodologies and decision-making tools may help adolescents and young adults express their thoughts and feelings about hope [44, 45]. Future work should evaluate strategies for elevating patient voices and explore the impact of empowering patients to voice their hopes during clinical communication.

## Limitations

This study has several limitations. The research was conducted at a large academic pediatric cancer center, with a selection bias for families who seek experimental trials; this lived experience may influence how families engage in conversations around hope. Relatedly, the demographics of our study population reflected institutional norms, but the sample size was not sufficient to saturate themes stratified by demographic and clinical characteristics. Further investigation is needed to deepen understanding of how different illnesses, cultural beliefs, lived experiences, and communication dynamics shape how individuals articulate and respond to hope language. Specifically, participants who spoke languages other than English were not eligible for participation, which may influence findings in unknown ways; studies with expanded eligibility are ongoing to address this limitation. While qualitative research is not designed for generalizability, we believe that these findings have the potential for transferability to other pediatric serious illness populations. We did not interview patients or parents to understand their perceptions of specific hope language, and a study to address this gap is also ongoing. Finally, data collection and analysis did not capture nonverbal communication and future work could examine nonverbal expressions of hope to better understand approaches that convey hope through tone, timing, silence, or nonverbal cues.

## Conclusions

In summary, these findings highlight opportunities to improve how clinicians provide space for patients and families to express hope and exemplify the challenge oncologists face when balancing expressions of hope with information about disease progression and incurability. Future work should explore how strategies that empower patients, parents, and oncologists to articulate and affirm hope may strengthen prognostic communication, clarify next steps, and align treatment with goals of care.

## Supplementary Files

This is a list of supplementary files associated with this preprint. Click to download.

- UCHATHopeSupplementsfinal.docx

## Figures and Tables

**Table 1.** Participant Demographics.

Characteristics	*N* (%)
**Patients** (*n* = 30)	
Gender	
*Female*	15 (50%)
*Male*	15 (50%)
Race	
*American Indian/Alaskan Native*	1 (3%)
*Black*	8 (27%)
*White*	19 (63%)
*Other: Biracial*	2 (7%)
Ethnicity	
*Hispanic*	3 (10%)
*Non-Hispanic*	27 (90%)
Patient Age at Diagnosis	
*0–2 years*	4 (13%)
*3–11 years*	18 (60%)
*12–18 years*	8 (27%)
*19+ years*	0 (0%)
**Parents** (*n* = 30)*	
Gender	
*Female*	26 (87%)
*Male*	4 (13%)
**Pediatric Neuro-Oncologists** (*n* = 7)	
Gender	
*Female*	2 (29%)
*Male*	5 (71%)
Race	
*Middle Eastern*	1 (14%)
*Southeast Asian*	2 (29%)
*White*	4 (57%)
Ethnicity	
*Hispanic*	0 (0%)
*Non-Hispanic*	7 (100%)
Years in Clinical Practice	
*1–4 years*	0 (0%)
*5–9 years*	3 (43%)
*10–19 years*	3 (43%)
*20+ years*	1 (14%)

* Race and ethnicity are not shown. Parent race and ethnicity were identical to patient race and ethnicity for all parents who reported demographic data (*n*=16).

**Table 2.** Definitions of Dimensions and Themes in Hope Dialogue.

Dimension 1: Temporal Orientation	
Theme	Definition
Prospective Hope	Expressed desire for positive future outcomes
Retrospective Hope	Reflection on hopes (fulfilled or unfulfilled) from the past
Negated Hope	Ruling out certain possibilities, setting limits on possible outcomes in the present or future
Dimension 2: Desired Outcomes	
Theme	Definition
Hope for Cure	Full recovery, no evidence of disease
Hope for Remission	Temporary or sustained absence of cancer
Hope for Life Prolongation	Halt or slow disease progression
Hope for Symptom Management and Comfort	Reduction of pain, nausea, distress, peaceful, meaningful, and comfortable final days.

**Table 3.** Exemplary Quotes for Temporal Themes.

Theme	Exemplary Quotes
Prospective Hopes	Patient: “As long as it [cancer] doesn’t…instantly kill me, oh well, I just have cancer, I have to live with it…Okay, let’s talk about homecoming…I know I am not able to go to school, I just want to go to the part where I am going to be wearing my suit, because I am customizing my suit for myself for homecoming.”
	Parent (to child): “Yeah, one important thing is we are able to be with our family for the last week of June and that’s our hope, because you [patient] would like to go to the family reunion.”
	Patient (to oncologist): “That’s when we see all of our seventeen cousins…we do cooking, campfire.”
Retrospective Hopes	Oncologist: “I was hoping, we were all hoping, that we get a little bit more, he’s having a great quality of life, you’ve enjoyed the summer with him… but we’re sort of headed in one direction now… I was very hopeful seeing how well she was doing that we would have a good scan. ”
	Oncologist: “Well I was just hoping that we’d just slow the growth, especially seeing her like this, unfortunately 1 don’t have any good news…Part of the reason she is not having any symptoms is because [the tumor] is not obstructing anything but eventually it will start doing that.”
Negated Hopes	Oncologist: “Unfortunately, these tumors will recur, will come back after a while, unfortunately that’s their nature. I don’t know how soon or how late…It is almost certain that it’s going to come back…I do not believe, in my heart, that this is going to cure her from the cancer.”
	Parent: “When we came in August, you had hopes because of the gene. Other options because of the immunotherapy?”
	Oncologist: “I don’t think there’s anything right now… There’s certainly nothing out there where I’m like, oh yeah we totally got to do this maybe it’ll fix it.”

**Table 4.** Exemplary Quotes for Desired Outcome Themes.

Theme	Exemplary Quotes
Hope for Cure	Parent: “I’m very careful about what I read and where I read. A lot of times, I skip through the depressing stuff and say, well, what’s the research? I don’t look often, just once in a while. It’s interesting to see some of the things that are going on and think, you know, hopefully one day this will be an answer.”
	Oncologist: “I hope so. You know how we laugh at infections, hopefully in the future someone will be laughing at our practice. That’s what we’re hoping for.”
	Parent: “I want a cure. As simple as that, why quit? We’ve had this conversation before - that I’m not going to stop fighting for her. Those [are] my hopes: Time. Cure. You think I want her in a clinical trial just for the pain?”
Hope for Remission or Stability	Oncologist: “I don’t believe this may cure her - but at least we are hoping that it will prevent it from coming for as long as possible.”
	Parent: “This is good news. Stable is always good news, yeah?”
	Patient: “Yeah. The tumor is changing inside of it.”
	Parent: “It is.”
	Patient: “That could be, right now, that could be good or bad.”
	Parent: “We’ll just say right now it’s stable and that is good. We’re just gonna keep working off that. Yeah, that’s great.”
Hope for Life Prolongation and Meaningful Experiences	Oncologist: “Trying to be realistic, no sugar coating, … I would say at the most, it might buy us some time, but I cannot promise you that. But at best that’s what I’m hoping for.”
	Parent: “I was just hoping to buy enough time.”
Hope for Symptom Management and Comfort at End of Life	Oncologist: “The reason for giving her the palliative radiation is to give her some comfort because tumor in your spine does not cause the end of life; it just makes it very painful and prolongs it, the suffering that is caused. I think I would give her at least a few weeks of comfort that she’s feeling.”
	Oncologist: “So that’s why I wanted you to talk to [palliative care team] before you leave.”
	Parent: “Yeah, we were discussing it earlier. I didn’t think about it that way. You’re right, I don’t want to get home and something happens and I don’t know what to do.”

## Data Availability

Data supporting the findings of this study is provided in the text and tables of the manuscript as transcribed quotes. Source files, including audio files and transcripts, are not openly available due to reasons of sensitivity and confidentiality. The corresponding author will consider reasonable requests for additional data on an as needed basis. Data are located in controlled access data storage at St. Jude Children’s Research Hospital.
